# Quassia as a Cure for Cholera

**Published:** 1884-10-20

**Authors:** Henry B. Evans

**Affiliations:** Lond., Picton, Ontario


### QUASSIA AS A CURE FOR CHOLERA.

BY HENRY B. EVANS, M.D. LOND., PICTON, ONTARIO.

I think it was about the year 1860 a formidable epidemic of cholera broke out in Bengal. All the public works were stopped, and amongst others the building of the Inuma bridge over the Ganges, and it was here that the demonstration of this simple, but wonderful cure for cholera took place. When all remedies seemed to fail, and the number of deaths to be daily on the increase, it was determined to try an empirical remedy (where or how it arose is not known), which was done with astonishing success. In ten days one hundred and forty-four patients had succumbed to the disease, and it yet increased in pace and destructiveness The superintendent of the works determined to try the "quassia remedy," as it was called, and he was most successful. The medicine not only acted as a cure, but also as a preventive. Twenty-four cases (bad ones), which were inoculated after the patients had been attacked, recovered. More than six hundred coolies were inoculated and escaped the disease, although remaining in the infected district, and not a death took place within a week.

The plan of treatment is very simple: Make an incision in the left arm, until the appearance of blood; then drop into the cut four to five drops of the tincture of quassia. So soon as the blood coagulates bind the wound up with a strip of cloth and keep it moist. The patients were allowed only cold water and sherbet to drink. Sometimes a *mussuck* of cold water was thrown over the body and head, for the medicine is found to cause extraordinary heat in the body, and this refreshed the patients much. The treatment was endorsed by reliable journals, as the Allahabad Gazette and the Northwest Gazette. How is this? Did some simple Hindoo stumble on an apology for the hypodermic syringe of late years? Did he, in a rough way, anticipate the discovery of Koch, and send his antidote right into the blood,* to strangle the cholera microbes in their element? There is something strange, too, about this simple drug. It is strongly antiseptic. An infusion of it will keep longer than that of any other vegetable. Discovered by Quassia, a negro, in the West Indies, as a cure for the fevers of that climate, and named after him, it seems deserving of a more

*Unfortunately for this supposition, cholera microbes have not yet been discovered in the blood. Koch declares emphatically that they do not enter the blood, and that they cause their effects through local action on the intestinal mucous surface.—ED. THER. GAZETTE.

extended notice. A cup made of this wood retains the property of rendering water bitter in it by immediate contact, and we know that it is a fatal fly poison. I write this to the Gazette, knowing that it has an earnest and diligent array of readers, who, I am sure, if an opportunity offers, will investigate the action of this medicine in cholera and other kindred diseases, where it may be equally useful, both as a cure and prophylactic.—*Therapeutic Gazette.*

